# Comparing the Coronal Seal of Different Thicknesses of MTA with Gutta-Percha after Post Space Preparation

**DOI:** 10.1155/2015/708639

**Published:** 2015-04-23

**Authors:** Mohammad Frough Reyhani, Hamidreza Yavari, Negin Ghasemi, Saeed Rahimi, Mohammad Hosien Soroush Barhaghi, Hadi Mokhtari, Payman Sarikhani

**Affiliations:** ^1^Department of Endodontics, Dental Faculty, Tabriz University (Medical Sciences), Tabriz 5154613475, Iran; ^2^Dental and Periodontal Research Center, Dental Faculty, Tabriz University (Medical Sciences), Tabriz 5154613475, Iran; ^3^Department of Microbiology, Medical Faculty, Tabriz University (Medical Sciences), Tabriz 5154613475, Iran; ^4^Dental Faculty, Tabriz University (Medical Sciences), Tabriz 5154613475, Iran

## Abstract

*Introduction*. The aim of this study was to compare the coronal seal of different thicknesses of MTA with gutta-percha after post space preparation. *Materials and Methods*. A total of 50 maxillary central incisors randomly divided into 5 experimental groups (*n* = 8). After preparation of the root canals with step back technique, in groups 1 and 2, post spaces were prepared and 4 or 5 mm gutta-percha remained in the apical, respectively. In groups 3, 4, and 5, there was 1-2 and 3 mm MTA in the apical, respectively. The ten teeth serve as control groups. The teeth were connected to a bacterial microleakage evaluation system. The study period was 120 days and the samples were evaluated on ten-day intervals; Meier-Kaplan technique was used for estimation of the mean time of microleakage to occur. Log-rank test was used for comparison of microleakage. *Results*. Number of samples exhibiting microleakage in MTA was less than those of gutta-percha at all intervals. Means of days with no microleakage were maximum and minimum with 3 mm MTA and 4 mm gutta-percha, respectively. *Conclusion*. Obturation of root canals using the MTA technique provides a proper seal with the minimum thickness of MTA in teeth requiring post space preparation.

## 1. Introduction

The aim of root canal obturation is to achieve a hermetic corono-apical seal and inability to achieve a proper seal is one of the most common reasons for failures of endodontic treatment [1].

Endodontically treated teeth should be properly restored to replace coronal structures, restore function, and prevent reinfections [2]. In the majority of cases, endodontically treated teeth have inadequate remaining structure and require post placement to increase the retention of the coronal restoration [3]. In addition, the post protects the apical seal against bacterial contamination due to coronal leakage [4].

To prepare the post space, some obturation material should be removed from the root canal, which affects the apical seal [2]. The integrity of the apical seal depends on the amount of obturation material remaining within the root canal [3, 5–7]. Studies have shown that in teeth, in which less than 3 mm of gutta-percha has remained after post space preparation, there is a higher incidence of lesions compared to greater gutta-percha lengths and the best coronal seal is achieved when at least 6 mm of gutta-percha remains in the root canal [3, 7].

In cases in which the roots are short, it is difficult to leave an adequate amount of gutta-percha in the apical end of the root canal during post space preparation. Therefore, in such cases it is advisable to use materials that provide adequate seal at a minimum thickness. Mineral Trioxide Aggregate (MTA) might be used as a root canal obturation material due to its favorable antimicrobial activity, biocompatibility, and sealing ability [6, 8–10].

Based on the results of previous studies, the sealing ability of MTA is higher than that of gutta-percha at similar lengths [11–13]. However, no studies to date have evaluated teeth with root canals filled with orthograde MTA at thicknesses less than those of MTA plug (1, 2, and 3 mm), prepared for post placement, and compared MTA with gutta-percha. Therefore, the aim of the present study was to compare coronal microleakage of teeth prepared with 1, 2, and 3 mm MTA plugs and 4 and 5 mm gutta-percha in teeth with short roots prepared for post placement, using bacterial leakage technique.

## 2. Materials and Methods

### 2.1. Selection of Teeth and Preparation of Samples

Fifty central incisors with mature apices and straight roots, and without caries and cracks, were selected. After removal of all the soft tissues from the teeth, they were placed in 0.5 chloramine T solution until they were used for the purpose of the study. The tooth crowns were removed using a diamond disk (D&Z, Wiesbaden, Germany) to leave roots measuring 13 mm in length. The patency of the canals was confirmed by placing a #10 file (K-File, Maillefer-Ballaigues, Switzerland) and root canals were filed up to #60 file (K-File, Maillefer-Ballaigues, Switzerland) using the step back technique and 5.25% NaOCl solution (Pakshoma, Karaj, Iran) for irrigation of the root canals.

To remove the smear layer at the end of root canal preparation procedures, 5 mL of 17% EDTA (Meta Biomed, Chungbuk, Korea) was used for 1 minute, followed by 5 mL normal saline and 5.25% NaOCl solution (Pakshoma, Karaj, Iran). Distilled water was used as the final rinse.

The samples were divided into 5 experimental groups (*n* = 5) according to root canal obturation protocol. Two control groups were considered (*n* = 5).

### 2.2. Root Canal Obturation and Post Space Preparation

The root canals were obturated as follows.

*Group 1*. The root canals were obturated with gutta-percha (Gapa Dent, Germany) and AH 26 sealer (Maillefer, Dentsply, Konstanz, Germany) using the lateral compaction technique. After completion of the obturation procedure, a heat carrier was used to remove 9 mm of gutta-percha from the root canals to leave 4 mm of the filling material at the apical end of the root canals. Then the remaining gutta-percha was packed with a cold plugger.

*Group 2*. All the procedures were similar to those in group 1 but 8 mm of gutta-percha was removed with a heat carrier to leave 5 mm of gutta-percha at the apical end of each root canal.

*Group 3*. In this group, 1 mm of the apical end of the root canals was obturated with MTA (Dentsply, Konstanz, Germany). To this end, based on the manufacturer's instructions, 1 g of MTA powder was mixed with 0.32 g of its liquid. The resultant mix was placed in root canals using an MTA carrier and moved to the end of the root canal with a plugger so that only 1 mm of MTA was left at the apical end of the root canal. Then a wet cotton pellet impregnated with phosphate-buffered saline solution was placed in contact with MTA within the root canal for 24 hours.

*Group 4*. The procedural steps were similar to those in group 3 but 2 mm of MTA was placed at the apical end of the root canal.

*Group 5*. The procedural steps were similar to those in group 3 but 3 mm of MTA was placed at the apical end of the root canal.

Regarding preparation of control groups, the teeth used as positive controls were instrumented but not obturated to demonstrate the maximum bacterial leakage through the root canal system. The negative control teeth were instrumented, not obturated and thorough sealed externally with nail polish in order to check the method.

In order to evaluate the quality and depth of root canal obturation materials the teeth underwent radiographic examinations. All the external surfaces of the teeth were covered two coats of nail varnish except for 2 mm of the apical end. All the samples in all the groups were placed in an incubator at 37°C under 100% relative humidity for 24 h.

### 2.3. Evaluation of Leakage

**Figure 1:**
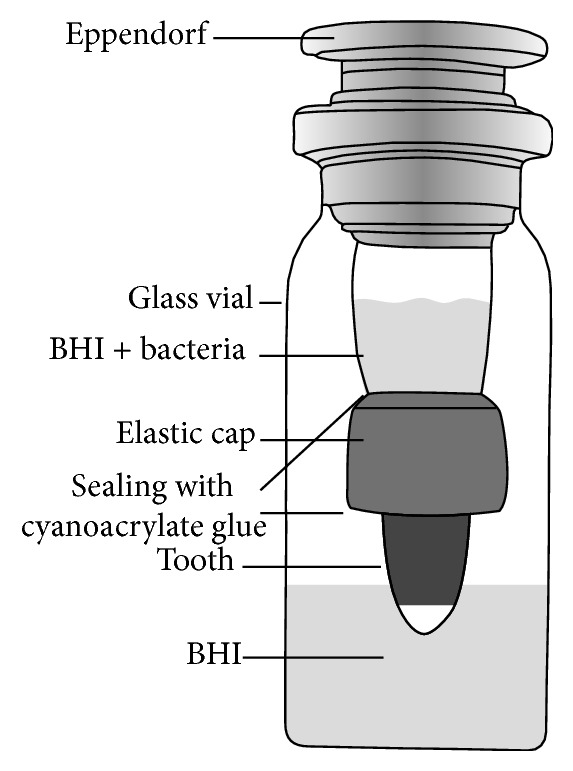
The leakage evaluation system.

In order to evaluate microbial microleakage, the samples were transferred to a kind of bacterial microleakage system (Figure 1). Then the Eppendorf tubes along with their teeth were passed through a hole in the distilled water vials that had been prepared. Then the contact point of the root and Eppendorf tube and that of the Eppendorf tube and the antiserum vial were sealed with cyanoacrylate glue (Evo-Band, Kaohsiung, Taiwan). This complex was sterilized with ethylene oxide gas for 24 hours. In the next stage, 15 mL of the BHI suspension with 1 × 10^8^ *E. faecalis* bacterial species (ATCC 29212) in each mL of it was injected into the upper chamber of the complex every three days, followed by incubation at 34°C for three days. Then microbial microleakage was evaluated by turbidity of the BHI suspension within the vial. The samples were evaluated daily for 120 days. When turbidity was observed the suspension was cultured on blood agar medium to make sure that bacterial contamination was caused only by* E. faecalis* species.

### 2.4. Statistical Analysis of Data

Kaplan-Meier method was used to calculate the mean of days with no microleakage. In order to evaluate absence of microleakage in the 5 groups, no-microleakage function curves were drawn. Log-rank test was used to compare no-microleakage functions curves. Statistical significance was set at *P* = 0.05. Statistical analysis was carried out by SPSS software (SPSS version 18.0, SPSS, Chicago, IL, USA).

## 3. Results

Table 1 presents the number of samples exhibiting microleakage in each group at each evaluation interval. It shows that in the samples under study the number of samples exhibiting microleakage with different thicknesses of MTA (1, 2, and 3 mm) was less than those of gutta-percha samples (4 and 5 mm) at all the time intervals.

Table 2 presents the mean days with no microleakage in each group. Based on the results presented in Table 2, it can be concluded that the means of days with no microleakage were maximum and minimum with 3 mm MTA and 4 mm gutta-percha, respectively.

The results of log-rank test showed a statistically significant difference in the days of no microleakage in the 5 study groups (*P* = 0.001). There were no statistically significant differences between no-microleakage functions of MTA (1, 2, and 3 mm) (*P* = 0.09), which held true in case of the two gutta-percha groups (*P* = 0.08).

The results of log-rank test showed significant differences between no-microleakage functions of MTA (1, 2, and 3 mm) and 4 mm gutta-percha (*P* < 0.05). Kaplan-Meier curves showed that the odds of persistence of no-microleakage state with MTA (1, 2, and 3 mm) were significantly higher than those of the two gutta-percha groups (*P* < 0.005).

## 4. Discussion

The aim of the present study was to compare microleakage of root canals obturated with orthograde MTA and gutta-percha at different thickness. The results showed superior sealing ability of 1, 2, and 3 mm thicknesses of MTA in comparison with 4 and 5 mm gutta-percha. On the other hand, bacterial microleakage of different thicknesses of MTA was not significantly different.

MTA is a bioactive material with various applications in endodontics, including its use as a root canal obturation material. The reasons for such an application of MTA include its bactericidal properties, its setting in the presence of blood and moisture, absence of shrinkage, and its sealing ability due to its chemical bond with the intracanal dentin [6, 14, 15]. A study by Martin et al. showed favorable sealing ability of MTA at 3–5-mm thicknesses in root canals obturated with orthograde MTA [13]. One of the advantages of the use of an MTA plug in teeth with short roots which are to receive posts is the fact that there is no need to remove root canal filling material from the canal and therefore no traumas are inflicted on the root canal walls during post space preparation [16]. However, with the use of gutta-percha, evacuation of gutta-percha might disrupt the root canal seal. One of the disadvantages of MTA is the difficulty of removing MTA when retreatment is required [17]. In the present study, the microleakage of MTA, as a root canal filling material, was less than that of gutta-percha which is a commonly used obturation material.

The ability of MTA to form a chemical bond with intracanal dentin [18] becomes important when an endodontically treated tooth with severe coronal destruction requires placement of an intracanal post for retention but has a short root, creating a limitation of space for the placement of a post with an ideal length. The ideal post length is 2/3 of the root length with bony support or at least equal to the root length. On the other hand, when gutta-percha is used for root canal obturation at least 4 mm of gutta-percha should remain at the apical end of the root canal after post space preparation, which is not possible in short roots [19]. In addition, in teeth with immature apices, which require post space preparation after endodontic treatment, use of MTA as a root-end filling material decreases procedural endodontic errors such as perforation or weakening of the thin root canal wall during removal of gutta-percha in addition to the use of bioactive properties of MTA [18, 20].

The integrity of the apical seal is proportionate with the thickness of the residual material [3]. Different techniques are available for the evaluation of the microleakage of root canal obturation materials, including due penetration, liquid diffusion, bacterial penetration and their endotoxins, radioisotope penetration, autoradiographs, and electrochemical techniques, each with advantages and disadvantages [21]. In the present study, microleakage was evaluated using the bacteriologic technique, which more closely simulates the clinical situation and is more reliable than the dye penetration technique because it is possible, with the application of this technique, to create artificial environments and evaluate the effect of bacterial penetration at very small scales.

In studies in which microbial microleakage is used some factors might affect microbial penetration, which include the technique used to prepare the teeth, the bacterial species used and its motility, and the antibacterial activity of the sealer and the root canal obturation material [19]. Several studies have shown differences between the results of bacterial leakage and dye penetration tests. Therefore, caution should be exercised in the interpretation of the results and their extension to clinical situations [21]. In the present study, the root lengths, the size of the master apical file, and the diameter of the coronal third of the root canal were standardized in all the samples so that a specific amount of bacteria would remain in contact with the root-end filling material in the post space [19]. In addition, long time intervals were included in the study because from a clinical point of view evaluation of the immediate seal of the material in the root canal is not useful and the material should provide a seal in the long run.

Different bacterial species have been used for the evaluation of microbial leakage in different studies. In the present study,* E. faecalis* was selected for bacterial microleakage assessment because it is a member of the oral flora and is usually isolated along with other aerobic and anaerobic bacteria in odontogenic infections and endodontically treated teeth which require retreatment due to root canal therapy failure [22]. In addition, this bacterial species can survive harsh conditions of the obturated root canal. It is highly resistant against the chemical and mechanical procedures during root canal treatment. Furthermore, it is resistant against a large number of antibacterial solutions [23]. On the other hand, in the present study only one bacterial species was used because in such a situation it is easier to interpret the results.

There are two important considerations in relation to leakage studies: the occurrence of microleakage and the time interval of such an occurrence [19]. In the present study, bacterial leakage occurred regardless of the time interval, consistent with the results of previous studies [3, 6, 9, 19]. Absence of any difference in the seal of 1, 2, and 3-mm thicknesses of MTA in the present study is consistent with the results of some previous studies using dye and bacterial penetration techniques to evaluate leakage [12, 15, 16]. However, as mentioned above, in the present study the aim was to achieve a proper seal with minimum material thicknesses, which can be applied in short and curved roots under similar clinical conditions. The higher sealing ability of MTA in the present study is consistent with the results of previous studies [3, 6, 20].

The results of the present study are not consistent with those reported by Valois and Costa and the discrepancy might be attributed to differences in the tests used; they used a protein solution microleakage test rather than bacterial microleakage test in their study [17]. They reported that the seal of 1-mm MTA thickness was less than that of 2 and 3-mm thicknesses.

## 5. Conclusion

Under the limitations of the present study, it can be concluded that obturation of root canals using the MTA provides a proper seal with the minimum thickness of MTA in teeth requiring post space preparation; so the clinicians can use one millimeter of MTA in special clinical situation in which application of proper thickness of gutta-percha was impossible. It is suggested that similar animal and clinical studies be carried out to achieve more accurate results that can be extended to clinical conditions.

## Figures and Tables

**Table 1:** The number of samples with microleakage in each group during the evaluation period.

Group	Size	Day 10	Day 20	Day 30	Day 40	Day 50	Day 60	Day 70	Day 80	Day 90	Day 100	Day 110	Day 120
MTA	1 mm			1	3	3	3	4	5	5	7	8	8
	2 mm		1	1	2	3	4	5	5	6	7	7	8
	3 mm			1	1	2	3	4	5	6	7	8	8

Gutta-percha	4 mm	1	4	6	8	8	8	8	8	8	8	8	8
	5 mm	1	3	5	7	8	8	8	8	8	8	8	8

Positive control	—	5	5	5	5	5	5	5	5	5	5	5	5

Negative control	—	0	0	0	0	0	0	0	0	0	0	0	0

**Table 2:** Estimation of means intervals of no microleakage in each group.

Material	Size	Estimate of the number of days with no microleakage
MTA	1 mm	67
	2 mm	64.3
	3 mm	75.7

Gutta-percha	4 mm	22.4
	5 mm	24.8

Positive control	—	10

Negative control	—	120
